# Retraction: Melatonin mediates osteoblast proliferation through the STIM1/ORAI1 pathway

**DOI:** 10.3389/fphar.2023.1299042

**Published:** 2023-09-28

**Authors:** Frontiers Editorial Office

The journal retracts the 2022 article cited above.

Following publication, the corresponding author contacted the editorial office stating that there are contributors that have been omitted from the authorship of the published article, despite providing substantial contributions that meet Frontiers authorship criteria. Furthermore, the corresponding author informed the editorial office about errors in data collection that affect the scientific validity of the article. It was found that the concerns were valid and that the article does not meet the standards of editorial and scientific soundness for Frontiers in Pharmacology, therefore, the article has been retracted.

This retraction was approved by the Chief Editors of Frontiers in Pharmacology and the Chief Executive Editor of Frontiers. The authors have agreed to the retraction.

